# Barriers to care for musculoskeletal sarcoma patients: a public health perspective

**DOI:** 10.3389/fpubh.2024.1399471

**Published:** 2024-08-21

**Authors:** Alina Syros, Max C. Baron, Jenna Adalbert, Hallie B. Remer, Marilyn Heng, Brooke Crawford

**Affiliations:** ^1^Harvard Combined Orthopaedic Residency Program, Harvard University, Boston, MA, United States; ^2^Department of Education, Miller School of Medicine, University of Miami, Miami, FL, United States; ^3^Department of Orthopedics, Miller School of Medicine, University of Miami, Miami, FL, United States

**Keywords:** access barriers in oncology, socioeconomic determinants of cancer care, public health, geographic barriers to care, musculoskeletal sarcoma, delays in care

## Abstract

**Introduction:**

This study seeks to investigate the barriers to care that exist for patients presenting with sarcomas of musculoskeletal origin. Understanding the roots of delays in care for patients with musculoskeletal sarcoma is particularly important given the necessity of prompt treatment for oncologic diagnoses. Investigators reviewed relevant studies of publications reporting barriers to care in patients undergoing diagnosis and treatment of musculoskeletal tumors.

**Methods:**

A comprehensive literature search was conducted using Scopus, Embase, Web of Science, and PubMed-MEDLINE. Twenty publications were analyzed, including a total of 114,056 patients.

**Results:**

Four barrier subtypes were identified: Socioeconomic Status, Geographic Location, Healthcare Quality, Sociocultural Factors. Socioeconomic status included access to health insurance and income level. Geographic location included distance traveled by patients, access to referral centers, type of hospital system and resource-challenged environments. Healthcare quality included substandard imaging, access to healthcare resources, and healthcare utilization prior to diagnosis. Sociocultural factors included psychological states, nutrition, education and social support.

**Conclusion:**

After identifying the most significant barriers in this study, we can target specific public health issues within our community that may reduce delays in care. The assessment of barriers to care is an important first step for improving the delivery of oncologic patient care to this patient population.

## 1 Introduction

Sarcomas are malignancies of the body’s connective tissue. They represent a subset of primary musculoskeletal cancers that account for less than 1% of all diagnosed cancers annually (1). These tumors are associated with significant morbidity and mortality and require prompt diagnosis and highly specialized treatment to achieve favorable outcomes (1). However, disparities in access to resources and inequitable distribution of care can create significant obstacles, resulting in delays in the delivery of healthcare services (2–4).

Numerous factors exist that may play a role in delayed patient presentation and can be broadly categorized by the five social determinants of health: economic stability, health care and quality, social and community context, neighborhood and built environment, and education (5, 6). These overlapping categories provide a context for understanding the barriers to care that exist in individuals with bone sarcoma. The authors believe that an in-depth understanding of the barriers to care for patients with bone sarcoma is particularly important given that without timely interventions, the disease may quickly progress beyond the limits of treatment (3, 4).

To the author’s knowledge, a systematic review of the barriers to care encountered by patients with musculoskeletal sarcoma has not been performed. Therefore, the aim of this study is to perform a high-quality systematic review of the literature to determine the most significant barriers to care that exist for patients undergoing diagnosis and treatment of musculoskeletal sarcoma.

## 2 Methods

### 2.1 Data source

Two experienced authors (HR, MB) conducted a comprehensive literature search using Scopus, Embase, Web of Science, and PubMed-MEDLINE databases from inception to June 6th, 2023. The search aimed to identify cohort studies, prospective and retrospective trials, randomized controlled trials, quasi-randomized control trials, and case series focusing on barriers to care for musculoskeletal sarcoma. The literature screening process was completed in accordance with Preferred Reporting Items for Systematic Reviews and Meta-Analyses (PRISMA) guidelines (Supplementary Figure 1). Database search terms included specific search phrases. Use of Boolean operators were as follows: (sarcoma) AND (orthopedics OR orthopedics OR musculoskeletal) AND (barriers OR obstacles OR challenges OR difficulties). Minor adjustments were made to the search phrase to accommodate the different databases. Any discrepancies were settled following the presentation to a senior author (JA; Supplementary Figure 1).

### 2.2 Study selection

Inclusion criteria were applied as follows: (1) full-text accessible; (2) published in English; (3) inclusion of multiple patients; (4) analyzed primary data (5) level 4 evidence or above; (6) investigation of barriers to care for musculoskeletal tumors. Exclusion criteria encompassed: (1) systematic reviews and meta-analyses; (2) individual case studies; (3) management of solely non-orthopedic cancers.

Initially, abstracts and titles were screened to retain only studies of musculoskeletal sarcomas which identified a barrier to optimal care for patients in diagnosis and/or treatment. Our definition of musculoskeletal sarcoma includes primary sarcoma of the bone, soft tissue, or connective tissue origin within the musculoskeletal system. Subsequently, two reviewers (HR, MB) independently extracted relevant data from each included full-text study such as author and publication year, study type, musculoskeletal tumor diagnosis, type of barrier to care, treatment course, and clinical outcomes if available (Table 1).

**Table 1:** Cited sarcoma studies: year, article type, journal, country, sample size, and cancer diagnosis.

Authors (Year)	Title	Article type	Journal	Country of origin	Sample size	Cancer diagnosis
Miller et al. (2017)	Socioeconomic measures influence survival in osteosarcoma: an analysis of the National Cancer Data Base	Retrospective Review	The International Journal of Cancer Epidemiology, Detection, and Prevention	USA	*N* = 3,505 n_Medicaid_ = 869 n_lowest quartile SES_ = 683	High-grade conventional osteosarcoma
Smartt et al. (2020)	Is There an Association Between Insurance Status and Survival and Treatment of Primary Bone and Extremity Soft-tissue Sarcomas? A SEER Database Study	Retrospective Review	Clinical Orthopedics and Related Research	USA	N_osteosarcoma_ = 4,144 n_non-Medicaid_ = 3,098 n_Medicaid_ = 884 n_uninsured_ = 162 N_soft tissue sarcoma_ = 7,508 n_non-Medicaid_ = 6,292 n_Medicaid_ = 904 n_uninsured_ = 312	Bone and soft tissue sarcomas
Jang et al. (2023)	Effect of Insurance Status on Mortality in Adults With Sarcoma of the Extremities and Pelvis: A SEER-Medicare Study	Retrospective Review	Journal of the American Academy of Orthopedic Surgeons	USA	*N* = 7,056 n_medicaid_ = 182 n_uninsured_ = 34 n_private ins_ = 1,533 n_medicare_ = 4,785	Bone and soft tissue sarcomas
Malik et al. (2021)	Has the Affordable Care Act Been Associated with Increased Insurance Coverage and Early-stage Diagnoses of Bone and Soft-tissue Sarcomas in Adults?	Retrospective Review	Clinical Orthopedics and Related Research	USA	*N* = 15,287 n_pre-ACA_ = 6,537 n_pre-Medicaid expansion_ = 5,076 n_post-Medicaid expansion_ = 3,674	Primary malignant bone tumors
Monsereenusorn et al. (2022)	Impact of treatment refusal and abandonment on survival outcomes in pediatric osteosarcoma in Southeast Asia: A multicenter study	Retrospective Review	Pediatric Blood & Cancer	Thailand	*N* = 208 n_TxRA_ = 59 n_non-TxRA_ = 149	Osteosarcoma
Wendt et al. (2019)	Rural patients are at risk for increased stage at presentation and diminished overall survival in osteosarcoma	Retrospective Review	Cancer Epidemiology	USA	*N* = 476 n_>2 h drive_ = 128	High Grade osteosarcoma
Fayet et al. (2022)	No Geographical Inequalities in Survival for Sarcoma Patients in France: A Reference Networks’ Outcome?	Retrospective Review	Cancers	France	*N* = 2,281 n_wealthy metropolitan area_ = 468 n_precarious district_ = 1,188	All Sarcomas: 6.8% bone, 69% soft-tissue, and 14.2% visceral sarcomas
Fayet et al. (2021)	Determinants of the access to remote specialized services provided by national sarcoma reference centers	Retrospective Review	BMC Cancer	France	*N* = 20,589	All Sarcomas: orthopedic and non-orthopedic sarcomas
Fujiwara et al. (2021)	Greater travel distance to specialized facilities is associated with higher survival for patients with soft-tissue sarcoma: US nationwide patterns	Retrospective Review	PLOS One	USA	*N* = 34,528 n_>101 miles_ = 2,143 n_51-100 miles_ = 2,761 n_11-50 miles_ = 12,729 n_<10 miles_ = 16,895	Soft tissue sarcoma
Sasi et al. (2023)	Determinants and impact of diagnostic interval in bone sarcomas: A retrospective cohort study	Retrospective Review	Pediatric Blood & Cancer	India	*N* = 1,227 n_osteosarcoma_ = 470 n_ewing sarcoma_ = 757	High grade Osteosarcoma, Ewing Sarcoma
Rædkjær et al. (2019)	Use of Healthcare Services Two Years before Diagnosis in Danish Sarcoma Patients, 2000–2013	Retrospective cohort study	Sarcoma	Denmark	*N* = 2,167 n_female_ = 972 n_male_ = 1,195 N_Reference Matched Cohort_ = 21,670	Soft tissue sarcoma and osteosarcoma
Schiavi et al. (2015)	Using a family history questionnaire to identify adult patients with increased genetic risk for sarcoma	Self Administered Sarcoma Clinic Genetic Screening (SCGS)	Current Oncology	Canada	*N* = 164 n_female_ = 102 n_female_ = 62	All Sarcomas
Weaver et al. (2020)	The complexity of diagnosing sarcoma in a timely manner: perspectives of health professionals, patients, and carers in Australia	Exploratory Qualitative Research Design	BMC Health Service Research	Australia	*N* = 60 n_health prof working w/ sarcoma pts_ = 21 n_pts diagnosed w/ sarcoma_ = 22 n_caregivers for ppl diagnosed w/ sarcoma_ = 17	Sarcoma
Dahan et al. (2017)	Proximal femoral osteosarcoma: Diagnostic challenges translate into delayed and inappropriate management	Retrospective Review	Orthopedics & Traumatology: Surgery & Research	France	*N* = 12 n_incorrect imaging_ = 2	Proximal femur osteosarcoma
Poudel et al. (2017)	Factors associated with local recurrence in operated osteosarcomas: A retrospective evaluation of 95 cases from a tertiary care center in a resource challenged environment	Retrospective Review	Journal of Surgical Oncology	India	*N* = 95 n_local recurrence_ = 15 n_no local recurrence_ = 80	Osteosarcoma
Hewitt et al. (2019)	Patient Perceptions of the Impact of Treatment (Surgery and Radiotherapy) for Soft Tissue Sarcoma	Single, Semi-structured Interviews	Sarcoma	United Kingdom	*N* = 19	Soft tissue sarcoma
Sasaki et al. (2018)	Validation of Different Nutritional Assessment Tools in Predicting Prognosis of Patients with Soft Tissue Spindle-Cell Sarcomas	Retrospective Review	Nutrients	Japan	*N* = 103 n_death within 1 yr_ = 15 n_1 yr survival_ = 88	Soft tissue spindle-cell sarcomas
Alamanda et al. (2014)	Effect of marital status on treatment and survival of extremity soft tissue sarcoma	Retrospective Review	Annals of Oncology	USA	*N* = 7,384 n_single_ = 2,977	Soft tissue sarcoma
Alamanda et al. (2015)	Racial Disparities in Extremity Soft-Tissue Sarcoma Outcomes A Nationwide Analysis	Retrospective Review	American Journal of Clinical Oncology	USA	*N* = 7,225 n_African American_ = 825	Soft tissue sarcoma
Siddiqui et al. (2015)	Neglected orthopedic oncology--Causes, epidemiology and challenges for management in developing countries	Retrospective Review	Indian Journal of Cancer	India	*N* = 18 n_low SES_ = 15 n_uneducated_ = 17	Bone and soft tissue sarcomas

### 2.3 Data extraction

No advanced statistical analyses could be performed due to the heterogeneity of study populations and differences in inquiry regarding specific barriers to care among the selected manuscripts.

## 3 Results

From a total of 1,504 publications initially screened, 20 articles met the eligibility criteria and were included in the final analysis. These articles comprised a collective sample of 114,056 patients. The included articles were further categorized into four distinct barrier subtypes as defined by the authors: socioeconomic status, geographic location, healthcare quality, and sociocultural factors. Five articles met the inclusion criteria for each barrier subtype.

### 3.1 Socioeconomic status

Five articles (25%) reported on barriers relating to socioeconomic barriers in musculoskeletal sarcoma care (Table 2).

**Table 2:** Insights into socioeconomic barriers from five key studies.

Study	Identified barrier	Sample size	Reported outcomes
Miller et al., 2017 (Cancer Epidemiol)	Insurance status & SES	*N* = 3,505 n_Medicaid_ = 869 n_lowest quartile SES_ = 683	Lowest quartile SES (HR 1.23) & Medicaid insurance (HR 1.18) predictors of decreased survival at 5 years
Smartt et al., 2020 (Clin Orthop Relat Res)	Insurance status	N_osteosarcoma_ = 4,144 n_non-Medicaid_ = 3,098 n_Medicaid_ = 884 n_uninsured_ = 162 N_soft tissue sarcoma_ = 7,508 n_non-Medicaid_ = 6,292 n_Medicaid_ = 904 n_uninsured_ = 312	Medicaid insurance had reduced survival than did patients with non-Medicaid insurance in both osteosarcoma and soft tissue sarcoma (HR 1.3, *p* = 0.003 & HR 1.2, *p* = 0.019) Uninsured patients had reduced survival with extremity soft-tissue sarcomas (HR 1.6, *p* = 0.001)
Jang et al., 2023 (J Am Acad Orthop Surg)	Insurance status	*N* = 7,056 n_medicaid_ = 182 n_uninsured_ = 34 n_private ins_ = 1,533 n_medicare_ = 4,785	Medicaid insurance as the primary insurer had a 28% higher mortality compared to private insurance (HR 1.28, *p* = 0.026) Medicare as the primary insurer showed no significant difference in mortality compared to private insurance (HR, 1.06, *p* = 0.243)
Malik et al., 2021 (Clin Orthop Relat Res)	Insurance legislation	*N* = 15,287 n_pre-ACA_ = 6,537 n_pre-Medicaid expansion_ = 5,076 n_post-Medicaid expansion_ = 3,674	Post Medicaid expansion, the proportion of early stage primary bone sarcomas increased (*p* < 0.001), and the proportions of late-stage cancers decreased (*p* < 0.001)
Monsereenusorn et al., 2022 (Pediatr Blood Cancer)	Predictive factors (SES) for treatment refusal and abandonment (TxRA)	*N* = 208 n_TxRA_ = 59 n_non-TxRA_ = 149	Income classification of countries in Southeast Asia (Philippines: *p* < 0.002, Singapore: *p* < 0.025) was associated with greater TxRA

Among the articles, four focused on the impact of insurance on musculoskeletal sarcoma care. Miller et al. (7) found that Medicaid insurance was associated with a hazard ratio (HR) of 1.18, indicating reduced survival rates in various types of sarcomas when compared to private insurance (7). Similarly, Smartt et al. reported HR values of 1.3 (*p* = 0.003) for osteosarcoma and 1.2 (*p* = 0.019) for soft tissue sarcoma for Medicaid insurance, further supporting the association with poorer outcomes (8). Consistent with these findings, Jang et al. revealed a HR of 1.28 (*p* = 0.026) for Medicaid insurance, indicating a higher mortality rate (3). Additionally, uninsured patients faced poorer outcomes when compared to patients with non-medicaid insurance, as observed in Smartt et al. study (HR 1.6, *p* = 0.001) (8).

Furthermore, Malik et al. explored the effect of insurance legislation on cancer staging and identified a positive impact following Medicaid expansion, with an increase in early-stage primary bone sarcomas (*p* < 0.001) and a decrease in late-stage cancers (*p* < 0.001) (9).

In terms of socioeconomic factors, Monsereenusorn et al. investigated treatment refusal and abandonment in osteosarcoma patients and discovered that lower income classifications in Southeast Asian countries were associated with higher rates of such occurrences (*p* < 0.002 for Philippines and *p* < 0.025 for Singapore) (3). Additionally, Miller et al. (7) also found that the lowest quartile Socioeconomic Status (SES) was a predictor of decreased 5-year survival (HR 1.23) (7).

### 3.2 Geographic location

Five articles (25%) examined barriers related to geography or locational factors in the context of musculoskeletal sarcoma care (Table 3). These studies explored the impact of access to regional referral centers, geographic regions, distance traveled, and resource-challenged environments on diagnosis, outcomes, and patient experiences.

**Table 3:** Exploring geographic barriers in musculoskeletal sarcoma care: findings from five studies.

Study	Identified barrier	Sample size	Reported outcomes
Wendt et al., (Cancer Epidemiol)	Access to regional referral centers	*N* = 476 n_>2 h drive_ = 128 did not describe how many counted as rural	Patients with >2-h drive to comprehensive cancer center showed increased incidence of metastases (*p* = 0.021) Rural status associated with increased mortality when controlling for size of the tumor (HR 1.58, *p* = 0.037)
Fayet et al., 2022 (Cancers)	Geographic region	*N* = 2,281 n_wealthy metropolitan area_ = 468 n_precarious district_ = 1,188	Precarious population districts associated with lower survival (HR 1.23) however no significant association in survival after adjustment for the clinical variables (HR 1.03)
Fayet et al., 2021 (BMC Cancer)	Rural region & social deprivation	*N* = 20,589	Patients who are the farthest from reference centers have a reduced likelihood of early access to specialized diagnosis [OR 1.18, 95% CI 1.06 to 1.31] and MTB discussion [OR 1.24, 95% CI 1.10 to 1.40]. However, the impact of distance is relatively small compared to clinical factors and previous research on accessing cancer-specialized facilities.
Fujiwara et al., 2021 (PLoS One)	Distance traveled to academic/research center	*N* = 34,528 n_>101 miles_ = 2,143 n_51-100 miles_ = 2,761 n_11-50 miles_ = 12,729 n_<10 miles_ = 16,895	Distance >100 miles traveled associated with greater overall survival compared to distance <10 miles traveled (HR 0.877, *p* < 0.001) Diagnosis at an academic/research institution associated with greater overall survival (HR 0.857)
Sasi et al., 2023 (Pediatr Blood Cancer)	Resource-challenged environment	*N* = 1,227 n_osteosarcoma_ = 470 n_ewing sarcoma_ = 757	Distance greater than 100 km was a predictor of a longer diagnostic interval (>4 months; *p* < 0.04) Place of residence did not impact the proportion of patients with good necrosis post neoadjuvant chemotherapy (*p* = 0.30)

Wendt et al. revealed that patients with over a 2-h drive to comprehensive cancer centers had increased incidence of metastases and higher mortality rates (*p* = 0.021) (10). Rural status was also associated with increased mortality, independent of tumor size (HR 1.58, *p* = 0.037). Additionally, Sasi et al. explored the impact of resource-challenged environments on diagnostic intervals and treatment outcomes in high-grade osteosarcoma and Ewing sarcoma patients. They discovered that a distance greater than 100 km was a predictor of a longer diagnostic interval (*p* < 0.04), while place of residence did not significantly impact the proportion of patients with good necrosis post neoadjuvant chemotherapy (*p* = 0.30) (11).

Contrasting results were found when Fujiwara et al. investigated the effect of distance traveled to academic/research centers on overall survival in soft tissue sarcoma patients (12). Their findings revealed that traveling more than 100 miles was associated with greater overall survival (HR 0.877, *p* < 0.001), and diagnosis at an academic/research institution was also linked to improved outcomes (HR 0.857).

Fayet et al. investigated the influence of geographic regions on sarcoma survival. They found that living in precarious population districts was initially associated with lower survival rates (HR 1.23), but this association became non-significant after adjusting for clinical variables (HR 1.03). Another study by Fayet et al. explored the impact of rural regions and social deprivation on access to specialized diagnosis [Odds Ratio (OR) 1.18, 95% Confidence Interval (CI) 1.06 to 1.31] and multidisciplinary tumor board (MTB) discussions [OR 1.24, 95% CI 1.10 to 1.40] (13). Patients residing farther from reference centers had reduced likelihoods of early access to specialized diagnosis and MTB discussions. However, the influence of distance was relatively small compared to clinical factors and previous research on accessing cancer-specialized facilities.

### 3.3 Healthcare quality

Five articles (25%) examined barriers relevant to the diagnostic period of musculoskeletal sarcomas (Table 4). These studies focused on various factors impacting timely diagnosis, including healthcare utilization prior to diagnosis, access to genetic questionnaires, delays in diagnosis, substandard imaging, and resource-challenged environments.

**Table 4:** Insights into diagnostic barriers in musculoskeletal sarcoma: key findings from five studies.

Study	Identified barrier	Sample size	Reported outcomes
Rædkjær et al., 2019 (Sarcoma)	Utilization of healthcare prior to diagnosis	*N* = 2,167 n_female_ = 972 n_male_ = 1,195 N_Reference Matched Cohort_ = 21,670	Sarcoma patients had significantly increased incidence rate ratios (IRR) in use of healthcare services compared to the matched cohort a year before their diagnoses. The IRRs were statistically significant during the 12 months leading up to the diagnosis, reaching its highest point in the last month with an IRR of 13.89 (CI 12.41–15.54). No significant differences in length of increased consultation rates between sarcoma type, stage, and grade
Schiavi et al., 2015 (Curr Oncol)	Access to genetic questionnaires	*N* = 164 n_female_ = 102 n_female_ = 62	A family history of cancer (up to 3rd-degree relatives) was reported in 69% patients diagnosed with a sarcoma. The SCGS questionnaire was valuable in identifying sarcoma patients who may benefit from a genetic assessment. By using this tool, one can identify families who meet the criteria for LFL gene evaluation.
Weaver et al., 2020 (BMC Health Serv Res)	Delay of Diagnosis	*N* = 60 n_health prof working w/ sarcoma pts_ = 21 n_pts diagnosed w/ sarcoma_ = 22 n_caregivers for ppl diagnosed w/ sarcoma_ = 17	Delays in diagnosis were associated with the limited availability of health services, lack of prompt referrals to a sarcoma specialist, and diagnostic challenges
Dahan et al., 2017 (Orthop Traumatol Surg Res)	Substandard imaging	*N* = 12 n_incorrect imaging_ = 2	Management was inappropriate in 2 (17%) patients. Patients did not undergo all the recommended imaging studies prior to surgery. Subsequently 1 of the 2 patients experienced a local recurrence and metastases after 6 years and died 1 year later.
Poudel et al., 2017 (J Surg Oncol)	Resource challenged environments of the developing world	*N* = 95 n_local recurrence_ = 15 n_no local recurrence_ = 80	More patients who had undergone a previous biopsy procedure outside of the home institution were found to have local recurrence (LR) compare to no local recurrence (NLR; *p* = 0.05) The mean delay in biopsy of the NLR group was 4.16 ± 4.81 weeks compared to 9.46 ± 6.5 weeks in the LR group (*p* = 0.0002)

Rædkjær et al. and Weaver et al. both identified delays in diagnosis as a significant barrier. Rædkjær et al. emphasized the increased utilization of healthcare services by sarcoma patients leading up to their diagnoses. They found that sarcoma patients had significantly higher incidence rate ratios of healthcare service use compared to a matched cohort during the year leading up to their diagnoses. The highest incidence rate ratio was observed in the last month prior to diagnosis with an IRR of 13.89 (CI 12.41–15.54) (14). Weaver et al. conducted exploratory qualitative research on factors contributing to delays in sarcoma diagnosis (4). Limited availability of health services, lack of prompt referrals to sarcoma specialists, and diagnostic challenges were identified as barriers associated with delays in diagnosis.

Schiavi et al. and Dahan et al. focused on specific aspects of the diagnostic process. Schiavi et al. identified that a family history of cancer (up to 3rd-degree relatives) was reported in 69% patients diagnosed with a sarcoma, and highlighted the importance of access to genetic questionnaires in identifying patients who may benefit from genetic assessment (15). On the other hand, Dahan et al. discussed the consequences of substandard imaging practices and how 1 of the 2 patients who underwent inappropriate imaging experienced a local recurrence and metastases after 6 years and died 1 year later (16).

Poudel et al. observed that patients who had undergone a previous biopsy procedure outside of the home institution had a higher incidence of local recurrence (*p* = 0.05) (17). Additionally, the mean delay in biopsy was significantly longer in the group with local recurrence compared to the group with no local recurrence (*p* = 0.0002).

### 3.4 Sociocultural factors

Five articles (25%) examined sociocultural barriers to care in patients with musculoskeletal sarcoma (Table 5). These studies shed light on various aspects of sociocultural factors that influence the patient experience and outcomes. Among these articles, one study specifically focused on how psychological states and mental health could manifest as barriers to care.

**Table 5:** Sociocultural barriers in musculoskeletal sarcoma care: insights from five key studies.

Study	Identified barrier	Sample size	Reported outcomes
Hewitt et al., 2019 (Sarcoma)	Psychological	*N* = 19	Interviews showed that concerns throughout treatment included lack of understanding of soft tissue sarcomas and apprehension about treatment plans. Further interviewers concluded that treatment could be perceived as being more bearable if social support networks are implemented
Sasaki et al., 2018 (Nutrients)	Access to nutrition	*N* = 103 n_death within 1 yr_ = 15 n_1 yr survival_ = 88	Higher Glasgow Prognostic Score, (p < 0.001), Geriatric Nutritional Risk Index (p < 0.001), and controlling nutritional (CONUT) score (p < 0.001) significantly differed between patients who died within 1 year and patients who lived longer. Higher Glasgow Prognostic Score (*p* < 0.004) is a risk factor for death within a year of diagnosis.
Alamanda et al., 2014 (Ann Oncol)	Marital status	*N* = 7,384 n_single_ = 2,977	Single status was associated with higher grade tumors (*p* = 0.013), less radiotherapy (*p* < 0.001), and fewer surgeries (*p* < 0.001). Single status found to be an independent predictor of sarcoma specific death (*p* < 0.0001)
Alamanda et al., 2015 (Am J Clin Oncol)	Race	*N* = 7,225 n_African American_ = 825	African American race was associated with larger tumor size (*p* < 0.001), less radiotherapy (*p* = 0.024), fewer surgeries (*p* = 0.002), and greater number of deaths (*p* < 0.001)
Siddiqui et al., 2015 (Indian J Cancer)	Education Status & SES	*N* = 18 n_low SES_ = 15 n_uneducated_ = 17	Causes of delay in seeking medical by patients of low education level and low SES was attributed to financial constraints, cultural and religious believes, and lack of access to health care facilities

Hewitt et al. conducted single, semi-structured interviews with patients diagnosed with soft tissue sarcoma (18). The interviews revealed that concerns throughout treatment included a lack of understanding of soft tissue sarcomas and apprehension about treatment plans. The study suggested that implementing social support networks could make treatment more bearable for patients.

Sasaki et al. conducted a retrospective review of patients with soft tissue spindle-cell sarcomas (19). Their findings highlighted the impact of access to nutrition on patient outcomes. Patients with a higher Glasgow Prognostic Score, (*p* < 0.001), Geriatric Nutritional Risk Index (*p* < 0.001), and controlling nutritional (CONUT) score (*p* < 0.001) had a significantly higher risk of death within 1 year of diagnosis. The study emphasized the importance of adequate nutrition in improving prognosis.

Alamanda et al. and Alamanda et al. both focused on sociocultural factors such as marital status and race in relation to the diagnosis and treatment of soft tissue sarcomas (20, 21). Alamanda et al. found that being single was associated with higher grade tumors (*p* = 0.013), less radiotherapy (*p* < 0.001), and fewer surgeries (*p* < 0.001). Single status was also identified as an independent predictor of sarcoma-specific death (*p* < 0.0001). In Alamanda et al.’s study, African American race was associated with larger tumor size (*p* < 0.001), less radiotherapy (*p* = 0.024), fewer surgeries (*p* = 0.002), and greater number of deaths (*p* < 0.001).

Siddiqui et al. explored the influence of education status and SES on the delay in seeking medical care among patients with bone and soft tissue sarcomas (22). Patients with low education levels and low SES experienced delays in seeking medical care due to financial constraints, cultural and religious beliefs, and lack of access to healthcare facilities.

## 4 Discussion

The present study documents the types of barriers to care encountered by musculoskeletal sarcoma patients. To the authors’ knowledge, this is the first study to systematically assess barriers to care by socioeconomic status, geographic location, healthcare quality, and sociocultural factors.

The major theme of these results emphasizes how low socioeconomic status (SES) constitutes the underlying common denominator and the most important barrier to care for this patient population. A multivariate analysis of the 2022 National Cancer Database demonstrated greater mortality of uninsured sarcoma patients in the first 2 years when compared to their insured counterparts (23). The association between insurance status and increased mortality was unchanged after adjusting for potential confounders, including disease stage at presentation (3, 24). Prior to the expansion of Medicaid in 2014 the mortality of uninsured sarcoma patients was 28% greater than their insured counterparts. Since the Medicaid expansion, we have seen a decrease in sarcoma mortality demonstrating the direct link between increased access to care and survivorship (3). This relationship may be partially explained by the fact that insurance status has been demonstrated to be a positive predictor of clinic attendance which can prevent delays in care and advanced-stage presentation of sarcoma (25, 26).

An additional barrier to care resulting in delays in diagnosis is the concept of distance decay in which the outcomes of oncology patients decrease the farther away they live from a referral center, as long-distance was associated with increased wait time for diagnostic investigations (25). Our study demonstrated that prolonged distance and rural status were associated with increased morbidity and mortality in some cases, but not all (27). The reasons for these contradictory findings are multifaceted and may include other confounders that lead to delays in diagnosis.

Delays in diagnosis are identified as a significant barrier, with factors such as limited availability of health services, lack of prompt referrals to specialists, and diagnostic challenges contributing to these delays. Substandard imaging practices were also highlighted as a concern, with adverse outcomes observed in patients who did not undergo recommended imaging studies before surgery. Access to genetic questionnaires was identified as crucial for identifying patients who may benefit from a genetic assessment. The studies conducted in resource-challenged environments further emphasized the impact of such settings on both the quality of care and outcomes for sarcoma patients.

Previous literature provides insights into the current management of bone sarcomas. Gutowski et al. emphasize the role of chemotherapy advancements in improving survival for bone sarcoma patients (28). Similarly, Böhm et al. also discuss differentiated treatment approaches for malignant primary bone tumors, such as osteosarcoma, chondrosarcoma, and Ewing’s sarcoma, which have shown notable improvements with adjuvant chemotherapy (29). If patients encounter barriers such as diagnostic delays, substandard imaging, limited access to genetic questionnaires, or resource-challenged environments, they may face difficulties in receiving these standardized treatments, resulting in poorer outcomes.

The results of our review corroborate previous literature which focused on the effect of healthcare quality on cancer outcomes. Moor et al. emphasized the significance of access to both cancer and general medical care for cancer survivors, by highlighting that survivors who did not receive necessary cancer care had lower education levels and higher rates of public or no insurance compared to those who received all required care (30). Arhi et al. demonstrated that delays in referral from primary care resulted in later-stage colorectal cancer diagnosis and worse prognosis (31). Furthermore, Aparicio et al. reveal that a substantial proportion (52%) of older adult patients with colorectal cancer receive sub-standard treatment (32). These results are similar to results we found in sarcoma with difficulty in diagnosis and sub-optimal imaging.

The barriers to care identified during the diagnostic period of musculoskeletal sarcoma include delays in diagnosis, substandard imaging practices, limited access to genetic questionnaires, and the impact of resource-challenged environments. Addressing these barriers is crucial to improving healthcare quality for sarcoma patients. Further research and improvements in healthcare systems are warranted to ensure timely diagnosis, appropriate imaging practices, and access to genetic assessment, particularly in resource-challenged environments.

The findings on sociocultural factors in musculoskeletal sarcoma care reveal the significance of addressing patient concerns, implementing social support networks, and ensuring access to adequate nutrition. These factors contribute to improved treatment experiences and patient outcomes. The influence of sociocultural factors such as marital status and race is evident, with single status associated with higher-grade tumors and poorer treatment outcomes, while African American race is linked to larger tumor size and increased mortality. Additionally, education status and socioeconomic status impact delays in seeking medical care due to various barriers. These findings highlight the importance of addressing sociocultural barriers to enhance sarcoma care and optimize patient outcomes.

The results from our review corroborate the existing literature underscoring the significance of sociocultural factors in cancer care. Haier et al. emphasize the implementation and modification of cancer care systems, particularly in low-and middle-income countries (LMICs) (33). Understanding and utilizing sociocultural incentives, such as free housing and access to education, are crucial in addressing resource challenges and improving care, especially for vulnerable populations like metastatic cancer patients in LMICs. Ward et al. highlight disparities in cancer outcomes related to race/ethnicity and SES, with residents of poorer counties having higher death rates from cancer (34). Additionally, even when accounting for poverty rates, African American, American Indian/Alaskan Native, Asian/Pacific Islander men, and African American and American Indian/Alaskan Native women, had lower five-year survival rates compared to non-Hispanic Whites. Alcindor et al. emphasize the importance of multidisciplinary team care at expert centers for sarcoma treatment (25). They report significantly improved oncologic outcomes for patients treated at high-volume centers. This finding suggests that access to specialized care and expertise plays a critical role in improving sarcoma treatment outcomes. Valencia et al. observe excessive mortality risk among BIPOC (Black, Indigenous, and People of Color) individuals compared to non-Hispanic White counterparts and note disparities in engagement in routine cancer screenings, treatment initiation, surgical interventions, and higher mortality rates within 5 years of diagnosis (35).

Although this paper provides an enhanced understanding to barriers to care in patients diagnosed with musculoskeletal sarcoma, the present study is not without limitations. As with many systematic reviews, this study is susceptible to biases including publication and selection biases. Further, there was a lack of homogeneity in the reporting of variables including healthcare quality measures, healthcare costs, sociocultural demographics, and geographic measures. This is likely secondary to the lack of prospective studies on the topic. For this reason, we were unable to perform high power statistical analyses, limiting the ability to critically appraise and draw comparisons from the published studies to date. In spite of the aforementioned shortcomings, the present study is the first to provide a comprehensive summary and evaluation of the most pressing barriers to diagnose and treatment of sarcomas of musculoskeletal origin. Future researchers should be focused on developing measurement tools and questionnaires that are widely accessible to more efficaciously capture factors that influence the care of patients with sarcomas of musculoskeletal origin.

The findings on sociocultural factors in musculoskeletal sarcoma care provide insights into the patient experience and outcomes. The identified barriers to care call attention to the need for social support networks, secure access to adequate nutrition, address disparities based on marital status and race, and improve healthcare access for individuals with low education levels and low SES. By addressing these barriers, healthcare systems can strive for more equitable care and enhance treatment outcomes for patients with sarcoma.

## 5 Conclusion

Given the variety of barriers that exist for patients with musculoskeletal sarcoma, key initiatives related to increasing accessibility to care within specific patient communities may reduce delays to care for oncologic patients. These barriers to care highlight the importance of public health initiatives focused on improving patient access based on both internal and external patient factors. Further studies are warranted to explore specific interventions that improve patient access and prevent sarcoma progression to an untreatable or complex surgical stage.

## Data Availability

The original contributions presented in the study are included in the article/Supplementary material, further inquiries can be directed to the corresponding author.
